# Suicide and Drug Overdose Mortality Among Washington State Workers: A Stratified Analysis by Industry, Occupation, Sex, and Race/Ethnicity, 2014–2023

**DOI:** 10.3390/ijerph23060699

**Published:** 2026-05-25

**Authors:** Luke W. Sampson, David K. Bonauto, Jennifer L. Marcum

**Affiliations:** Washington State Department of Labor and Industries, Olympia, WA 98501, USA; david.bonauto@lni.wa.gov (D.K.B.); jennifer.marcum@lni.wa.gov (J.L.M.)

**Keywords:** industry, occupation, suicide, drug overdose, Washington state, construction, agriculture, accommodation and food services, farming, disparities

## Abstract

**Highlights:**

**Public health relevance—How does this work relate to a public health issue?**

- Suicide and drug overdose deaths are main contributors to overall mortality among the working-aged population.
- The industry and occupation someone works in can greatly impact health outcomes.

**Public health significance—Why is this work of significance to public health?**

- Previous research does not define the burden of suicide and drug overdose mortality while accounting for racial/ethnic and sex distribution within industry and occupational groups.
- Understanding the suicide and drug overdose risk related to industry and occupation, rather than the risk associated with age, race/ethnicity, or sex, is critical in identifying industries and occupations where workplace factors may be influencing risk.

**Public health implications—What are the key implications or messages for practitioners, policy makers, and/or researchers in public health?**

- Better understanding the effect of industry and occupation on suicide and drug overdose risk may lead to more impactful workplace suicide and drug overdose prevention programs.
- This research can and should be replicated by other states to better understand the risk of suicide and drug overdose across industry and occupation, as well as confirm or deny the generalizability of our findings.

**Abstract:**

Even with the rise in concern over suicide and drug overdose mortality in the United States, gaps in research on at-risk populations still exist. The purpose of this study was to investigate disparities in suicide and drug overdose mortality risk among Washington State workers. Using 2014–2023 Washington State death records and American Community Survey (ACS) data, we calculated rates of suicide and drug overdose mortality by usual industry and occupation while adjusting for age and stratifying by sex and race/ethnicity. We compared the mortality risk among workers in specific industries and occupations to all workers within the same sex and race/ethnicity strata to understand how work differentially affects risk. Working in Construction & Extraction occupations was associated with an increased risk for suicide and drug overdose death for males across all race/ethnicity categories and for drug overdose death among White females. The suicide risk for Asian/Pacific Islander males had the largest increase—Asian/Pacific Islander males working in Construction & Extraction occupations had a rate 4.59 times higher than all Asian/Pacific Islander male workers. The Education, Training, & Library occupation group had significantly lower crude rates and rate ratios. Although the causal pathways that may lead someone to die by suicide or drug overdose are complex, understanding risk profiles among different industries and occupations may lead to more appropriate prevention strategies.

## 1. Introduction

The increase in suicide and drug overdose deaths in the United States has contributed to the declining life expectancy of Americans [1]. Suicide and drug overdose deaths have drawn sustained attention from economists, policy makers, and health officials alike. Unintentional drug overdoses and suicide from all means combined were the third and fifth leading causes of death, respectively, among working-aged adults (16–65 years) in the United States (US) from 2018 to 2023 [2]. Washington State ranks 24th for suicide and 9th for drug overdose mortality among the US states [3,4]. These causes of death among the working-aged population negatively affect communities and families. There are direct economic impacts from medical costs and loss of household income, as well as psychological and physical “ripple” effects associated with suicide bereavement on close contacts of the deceased [5,6,7,8].

Using the International Classification of Diseases (ICD) system for classifying mortality, suicides represent a group of deaths determined to be caused by intentional self-harm [9]. Suicides may be associated with different mechanisms of death, such as firearm or drug overdose. Alternatively, drug overdose deaths may be intentional (suicide or homicide), or of unintentional or undetermined intent. Misclassification of suicides is a well-documented limitation in assessing true burden and is often attributed to stigma related to religion or social acceptability [10,11]. Additionally, intent of drug overdose is especially challenging to determine. Bohnert and Ilgen describe intentionality for drug overdose to be “dimensional, rather than categorical,” while providing evidence that they appear on a continuum somewhere between unintentional and intentional [12]. Drug overdose deaths are typically recorded as unintentional unless a suicide note is found, which is in approximately one-third of overdose deaths [13].

Not only are these two categories of mortality linked in classification, but they also have a myriad of overlapping potential causal pathways, risk factors, and prevention approaches [12]. Opioid use has been identified as a strong predictor of both suicide and lethal overdoses. Lack of opportunity leading to “despair” and increased availability of opioids are both seen as potential causal pathways for the increase in both suicide and drug overdose mortality [1,14]. Mental health conditions and substance use disorders are risk factors highly associated with both causes of death, and interventions that address both suicide and drug overdose risk are common [12]. Because of these overlaps, we investigate both categories of mortality in our work.

Suicide and fatal drug overdose rates vary by age and sex, with working-aged men at especially high risk of both causes of death in the US [2]. Additionally, there are known differences by race. Risk for drug overdose deaths is highest among Black and White men, while suicide risk is highest among White, American Indian/Alaskan Native, and Asian/Pacific Islander men [2]. It is also important to consider industry and occupation because work and the workplace may contribute to risk and protection for suicide across the social-ecological model [15]. These demographic differences in suicide and overdose rates are important when considering the intersectionality of risk factors in historically marginalized groups [15,16]. The intersectionality of age, sex, race/ethnicity, and industry/occupation highlights how systemic inequalities and societal structures can create and exacerbate risk of suicide and overdose mortality [15,17].

Previous researchers have investigated disparities in suicide and drug overdose deaths among workers by industry and occupation [18,19,20,21]. Differences in workplace culture and stigma, job precarity, and physical demands that increase potential use of opioids from chronic pain are cited as potential industry/occupation-specific risk and protective factors that could affect suicide and overdose mortality risk [18,19,22]. Occupations in Construction & Extraction and Farming, Fishing, & Forestry have been repeatedly cited to have higher suicide and fatal drug overdose risk compared to other occupations [18,19,20]. These occupations also tend to employ a high proportion of males, and males, as discussed above, are already at higher risk for suicide and drug overdose death [19,23,24]. Although there has been progress for increased diversity in employment across industry and occupational groups in the United States, there are still racial and gender disparities and occupational segregation in the U.S. labor force [22]. This relationship can complicate the effect of industry and occupation on suicide and drug overdose mortality risk. For example, are high rates among Construction & Extraction workers just a reflection of the risk among White, Non-Hispanic males, or is there additional risk related to the occupation itself? Studies must account for confounding and effect-modifying variables, e.g., race and ethnicity, that impact risk of suicide and drug overdose mortality and likelihood of working in a particular industry or occupation [25].

Previous studies of suicide and drug overdose risk by industry or occupation have accounted for some of the differences by age, sex, and race/ethnicity, though not comprehensively [18,19,21,26]. In many of the previous studies, race/ethnicity is treated as a confounder only, and authors present suicide or drug overdose rates that have been adjusted for the differences in race/ethnicity distributions among the workforce [18,19,21,26]. However, these adjusted death rates only present the disparities by industry and occupation while holding race/ethnicity constant. Adjustment alone for demographics may mask important demographic disparities within industries or occupations [25]. Therefore, we aimed to describe the differences in suicide and fatal drug overdose risk by sex and race/ethnicity within industry and occupation groups in Washington State to provide a deeper understanding of the relationship between risk and work. We compare the stratified rates within industry and occupation to the stratified rates of all adults in the labor force by sex and race/ethnicity to understand how working in certain industries and occupations may influence an individual’s baseline risk that is associated with their respective demographic groups (e.g., sex, race/ethnicity). In further understanding industries and occupations that have more or less correlation with these causes of mortality, efforts for suicide and drug overdose mortality reduction might consider workplace risk factors and bring forth prevention programs specific to the workplace [27,28,29]. We focus this study on Washington State to provide information that could be used for prevention program development at the state and local levels. We anticipate this work will also add to and confirm previous results that may be generalizable to other states.

## 2. Materials and Methods

### 2.1. Study Design

We designed a ten-year study to understand how work and other individual-level demographics may together influence risk of suicide and drug overdose mortality among non-institutionalized civilian Washington workers aged 16–65 years. Data were collected from Washington State death certificates and the American Community Survey (ACS) for the study period of 2014–2023 to calculate mortality rates while adjusting for age and stratifying by sex, race/ethnicity, industry, and occupation to minimize the potential bias introduced due to demographic segregation of industry and occupation. Industry describes the primary economic activity of the workplace, whereas occupation describes the work that a worker does, irrespective of where they are employed. We used both industry and occupation to characterize work, but included only select results by occupation in the main body of the manuscript for brevity. We provide detailed results for both industry and occupation in Tables S1–S8.

### 2.2. Washington Death Certificate Data

Death records were supplied to the Washington State Department of Labor and Industries via a data-sharing agreement with the Washington State Department of Health. We collected decedent demographics from the death records for analysis, including age at death, race/ethnicity, sex, industry, occupation, and state of residence. Suicide and fatal drug overdose cases were identified using the underlying cause of death codes, which are coded to the International Classification of Diseases, 10th (ICD-10) revision. Methods by Applebaum et al. (2019) were used to classify suicide deaths (ICD-10 codes: X60-X84, U03, Y870, Y10-Y33) and drug overdose deaths (ICD-10: X40-X44, X60-X64, X85, Y10-Y14) and account for the misclassification biases discussed earlier [30]. Deaths not coded as suicide and/or drug overdose are presented as “all other deaths”. To account for potential misclassification, suicide cases included deaths with intentional (93.9%) and undetermined (6.1%) intent, and fatal drug overdose cases included those coded as having intentional (7.3%), unintentional (90.8%), and undetermined intent (1.9%) [30].

“Usual” industry and occupation are collected as free-text information on death certificates. Structured data, however, was needed for analysis. Therefore, we used coded industry and occupation data provided by the National Institute for Occupational Safety and Health (NIOSH) National Occupational Mortality System (NOMS) for Washington State. Free text industry and occupation data are entered into the NIOSH Industry and Occupation Computerized Coding System (NIOCCS) from which Census industry and occupation codes are returned. The data used in this study were coded to the 2018 U.S. Census industry and occupation codes.

### 2.3. American Community Survey (ACS) Data

The US Census Bureau and the US Bureau of Labor Statistics annually conduct the American Community Survey (ACS) to collect information on housing and socioeconomic characteristics in the US. These data are weighted to be representative of the US population, and by state. The ACS was used in this study to estimate the number of civilian Washington residents aged 16–65 years old who reported being employed in the past 5 years (workers), by age, sex, race/ethnicity, industry, and occupation from 2014 to 2023. The ACS collects free-text industry and occupation from respondents, and responses are coded to 4-digit detailed Census industry and occupation codes. The ACS industry and occupation data describe the most recent employment from respondents for up to five years since their last employment. The ACS data were accessed using the IPUMS USA (formerly, the Integrated Public Use Microdata Series) website and database [31].

### 2.4. Study Population

**Figure 1:**
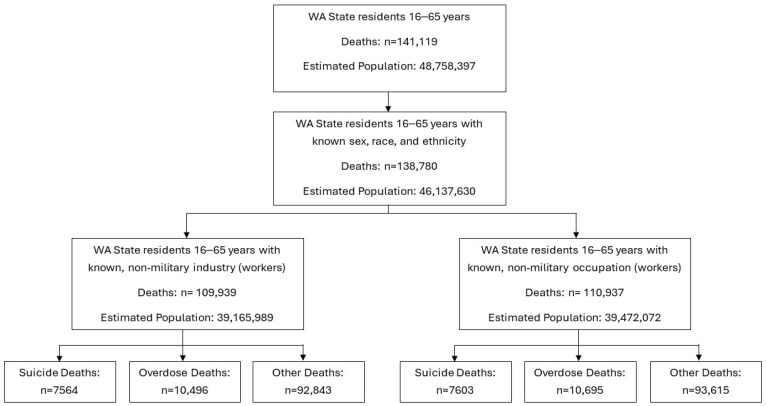
Flow chart of Washington State residents aged 16 to 65 years inclusion/exclusion criteria.

Figure 1 details the inclusion and exclusion criteria used in this study. Only death records and population estimates among Washington residents aged 16 to 65 years were collected for analysis. Washington residents were considered workers if they had any coded industry and occupation other than “unpaid worker”, “student”, “retired”, “disabled”, or “unknown”. This study focused on civilian workers, and so, military industries and occupations were excluded from industry- and occupation-specific analyses.

After the exclusions based on age and residency, 78% of the remaining decedents had known sex, race/ethnicity, and industry or occupation data; ~85% of the estimated working population remained to be used as denominator data for industry- and occupation- specific rate calculations. Since the decedents aren’t a direct subset of the estimated population, we assumed that the estimated working population industry and occupation reported from the ACS closely aligned with the usual industry or occupation reported on the decedents’ death certificate. This assumption is based on previous research by Luckhaupt et al., which reported ‘good’ to ‘very good’ alignment of usual and current industry and occupation, especially for broader categories of industry and occupation and among those aged below 65 [32].

### 2.5. Data Analysis

All rates were calculated using the death counts from the death certificate data as the numerator and weighted population estimates from the ACS as the denominator. Guidance from the National Center for Health Statistics was used to calculate 95% confidence intervals for vital rates with population denominator estimates [33]. The replicate weights provided by ACS were used in the variance calculations for the confidence intervals. Rates were suppressed from publication if the relative standard error (RSE) was greater than 25% [34]. Any count greater than zero and less than ten was suppressed for confidentiality of the deceased [34].

For analyses, we categorized the detailed industry and occupation codes from the death certificates and ACS into the National Health Interview Survey (NHIS) 2-digit ‘simple’ occupation and industry groupings to ensure numerator and denominator alignment [35]. The NHIS simple categories used in this analysis include 20 groups for industry and 22 groups for occupation--these roughly match the North American Industry Classification System (NAICS) industry sector codes and the Standardized Occupational Classification (SOC) major groups, respectively. Sex was limited to either “male” or “female” for numerator and denominator alignment. Race/ethnicity was combined into five mutually exclusive groups: Hispanic, all races combined; American Indian/Alaskan Native (AI/AN); Asian/Pacific Islander (Asian/PI); Black, or White.

Recent trends of suicide and drug overdose deaths among Washington workers were examined by calculating mortality rates for each year during the study period (2014–2023). Mortality rates were then calculated for the combined study period to examine risk of death by suicide and drug overdose for work characteristics (industry and occupation) and other individual-level demographics (age, sex, race/ethnicity). We stratified rates by sex, race/ethnicity, industry, and occupation and adjusted for age using direct standardization to the Washington state age group distribution [18]. We then compared the age-adjusted mortality rates within each demographic stratum to the same stratum working in each industry or occupation group by calculating rate ratios. These rate ratios describe how working within specific industries or occupations may differentially affect the baseline risk associated with demographics (sex and race/ethnicity). A rate ratio above one indicates the mortality risk is higher for a specific sex and race/ethnicity stratum working within the specific industry or occupation as compared to the risk associated with that demographic stratum alone, and a ratio below one indicates lower risk for the workers in that industry or occupation. We did not use formal statistical hypothesis testing to determine differences among adjusted rates. We assessed differences using both the point estimates and width of the 95% confidence intervals [36,37]. Rate ratios with 95% confidence intervals that do not include one are statistically significant at the α = 0.05 level [38]. All analyses were performed with SAS v9.4. The Washington State Institutional Review Board reviewed this study and determined it to be research involving publicly available records and non-living subjects, and thus, further review was waived.

## 3. Results

### 3.1. Crude Suicide and Drug Overdose Rates

**Figure 2:**
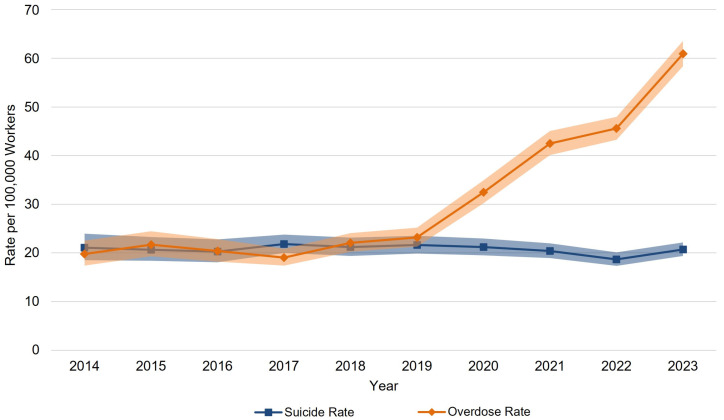
Crude suicide and drug overdose mortality rates and 95% confidence intervals among Washington residents by year, 2014–2023.

To understand recent trends of suicide and fatal overdose in Washington, crude mortality rates are provided by year from 2014 to 2023 (Figure 2). Suicide rates among this population remained level for the duration of the study period. Meanwhile, the rate of fatal drug overdoses increased by 174% since 2019 (Figure 2).

Suicide and drug overdose death rates by demographic variables are displayed in Table 1. The overall crude suicide and fatal drug overdose rates during 2014–2023 were 20.8 and 30.9 per 100,000 Washington (WA) residents, respectively (Table 1). Males had a higher risk of suicide (3.0× higher), fatal drug overdose (2.0× higher), and all other causes of death combined (1.5× higher) compared to females. Suicide rates were lower among those aged 16–44 years and increased among those aged 45 years or older, while drug overdose death rates increased with increasing age. Suicide and drug overdose death rates varied by race/ethnicity, with American Indian/Alaskan Natives (AI/AN) being at the highest risk for both types of death. Black WA residents had a relatively low rate of suicide, but high rate of drug overdose death. Interestingly, ‘all other’ death rates had similar mortality rate increases among American Indian/Alaskan Natives, Black, and White WA residents.

Crude rates of suicide varied across industries, from 6.9 per 100,000 WA workers in Education to 56.0 per 100,000 WA workers in Mining, Oil, & Gas (Table 2). Of note, Mining, Oil, & Gas is a relatively small industry, and the rates are based on small numbers (n = 23 suicides and n = 20 drug overdose deaths for the entire study period). Education had the lowest crude rate of drug overdose mortality, whereas the highest rate of drug overdose mortality was in Construction (5.2 and 84.1 per 100,000 workers, respectively).

There are similar patterns when we look at suicide rate by occupation, especially where there is considerable overlap between the industry and occupation groups. For example, workers in Construction & Extraction occupations had a relatively high suicide rate, Table 3, as did workers in the Construction industries. There are occupation groups, however, that work across several industries or industries that employ many occupation groups. For these instances, additional information on suicide risk among workers is apparent when grouped by occupation. For example, Installation, Maintenance, & Repair occupations are identified in Table 3 as a group of high-risk workers that would not have been distinguished if evaluated by industry alone. Protective Services occupations are another group of high-risk workers that would be missed if only evaluating industry, as they largely work in the Public Administration industry group.

### 3.2. Age-Adjusted Rates and Rate Ratios

**Figure 3:**
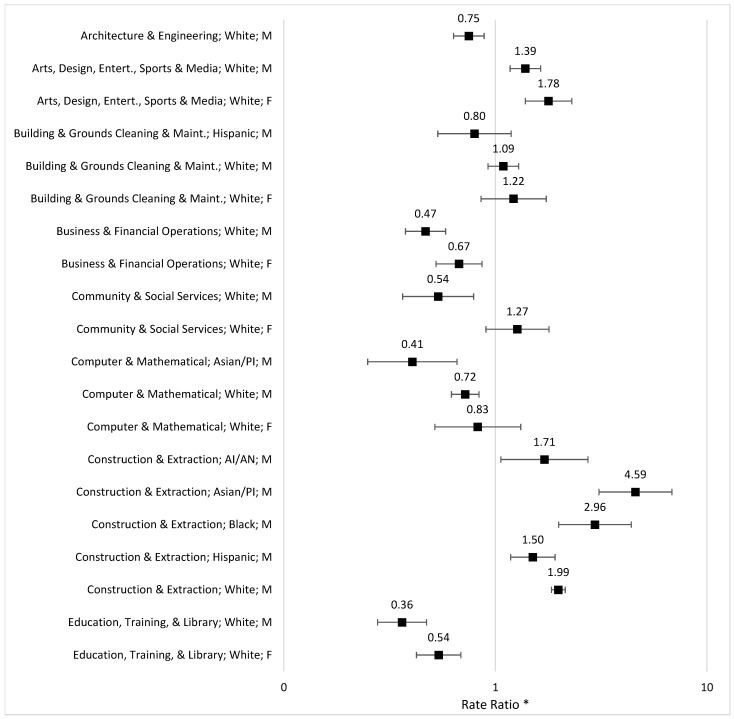

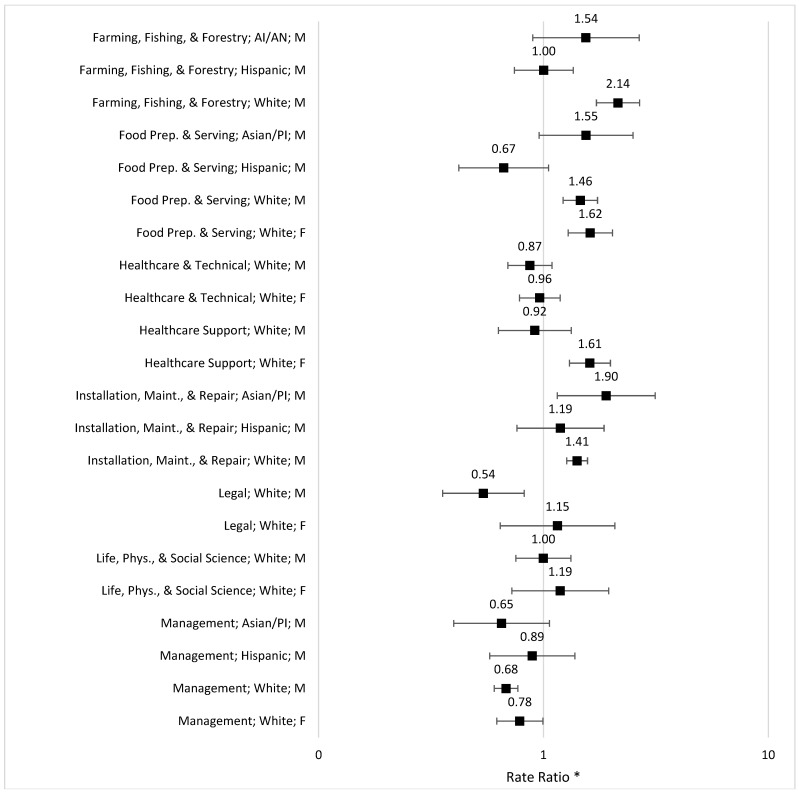

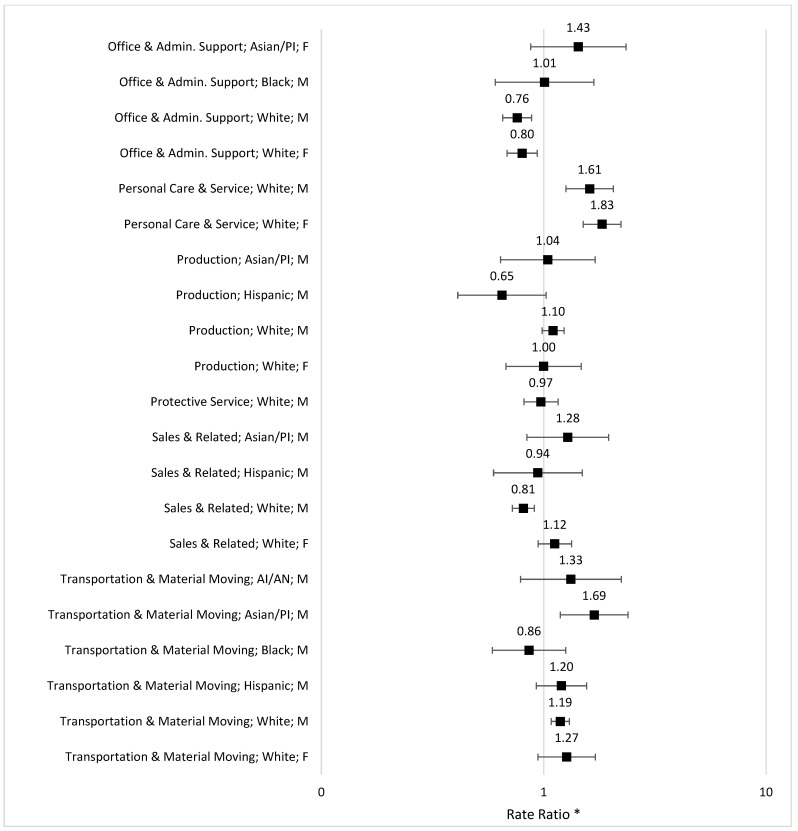
Suicide rate ratios and 95% CI for Washington workers stratified by occupation group, race/ethnicity, and sex, 2014–2023. * Suicide rate ratios comparing age-adjusted rates among workers in specific occupations to the baseline age-adjusted rate for all workers in the same race/ethnicity and sex group. 95% CI = 95% Confidence Interval; AI/AN = American Indian/Alaskan Native; PI = Pacific Islander.

All rates discussed below have been adjusted for age. Mortality rates for each stratum of sex, race/ethnicity, and simple industry and occupation groups are displayed in Tables S1–S8. For brevity, we discuss results by occupation only. Some strata have been suppressed due to small numbers and high RSEs. The highest suicide rates were among American Indian/Alaskan Native males working in Construction & Extraction (131.6 per 100,000); Farming, Fishing & Forestry (119.2 per 100,000); and Transportation & Material Moving (102.3 per 100,000) occupations, Table S3. This result is not surprising given the relatively high suicide rate among all male Native American/Alaskan Native WA residents, Table 1. When we compare the suicide rate of male Native American/Alaskan Native workers in a specific occupation to the baseline rate for that same demographic group working in any occupation, important new information about risk emerges. We provide rate ratios to illustrate the change in suicide risk associated with working in a specific group of occupations for each sex and race/ethnicity strata in Figure 3. Native American/Alaskan Native males working in Construction & Extraction occupations are 1.71 times more likely to die by suicide than Native American/Alaskan Native males working in all occupations combined, Figure 3. In other words, working in Construction & Extraction occupations further increased the risk of suicide in an already high-risk group. Working in the following occupation groups was associated with increased suicide risk among both male and female White workers as compared to their baseline– Arts, Design, Entertainment, Sports & Media; Food Preparation & Serving; and Personal Care & Service, Figure 3. Alternatively, working in Farming, Fishing, & Forestry or Transportation & Material Moving did not meaningfully change the suicide risk for Native American/Alaskan Native male workers, Figure 3. Working in the Construction & Extraction occupations was associated with increased suicide risk for males of each race/ethnicity group at varying amounts (Figure 3).

There were also occupation groups associated with decreased risk of suicide for certain race/ethnicity and sex groups. For example, White males and females working in Education, Training, & Library occupations had lower rates of suicide compared to the baseline rates for their respective race/ethnicity and sex groups. Some occupations only affected risk of suicide for certain demographic groups, not all. Working in Farming, Fishing, & Forestry occupations increased suicide risk by more than two times among White male workers, but did not meaningfully change the baseline risk among American Indian/Alaskan Native or Hispanic male workers. White females working in Healthcare Support occupations had an increased risk for suicide compared to the White female baseline, while White males working in those same occupations maintained their baseline risk. Earlier, we identified Protective Services workers as having an increased risk of suicide compared to all other workers (Table 3). When stratified by sex and race/ethnicity, however, White males working in Protective Services had the same suicide risk as compared to White males working in all occupations combined (Figure 3).

**Figure 4:**
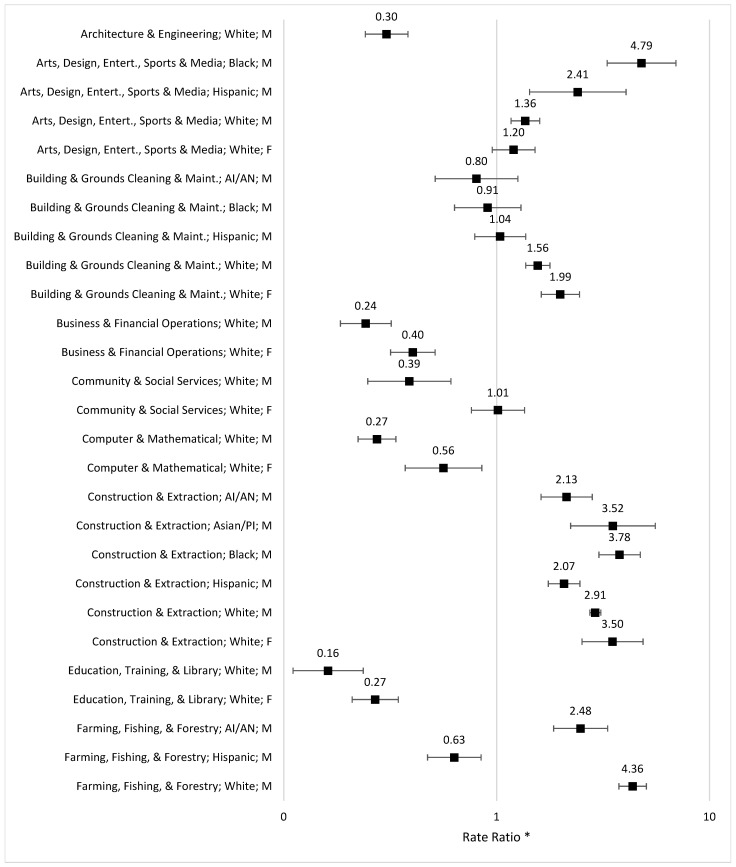

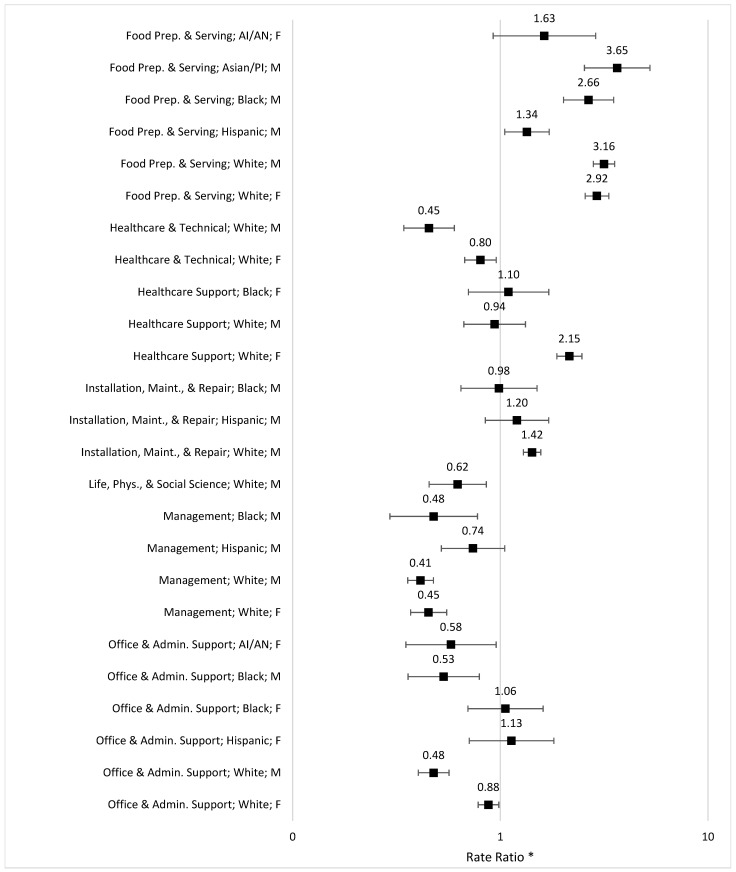

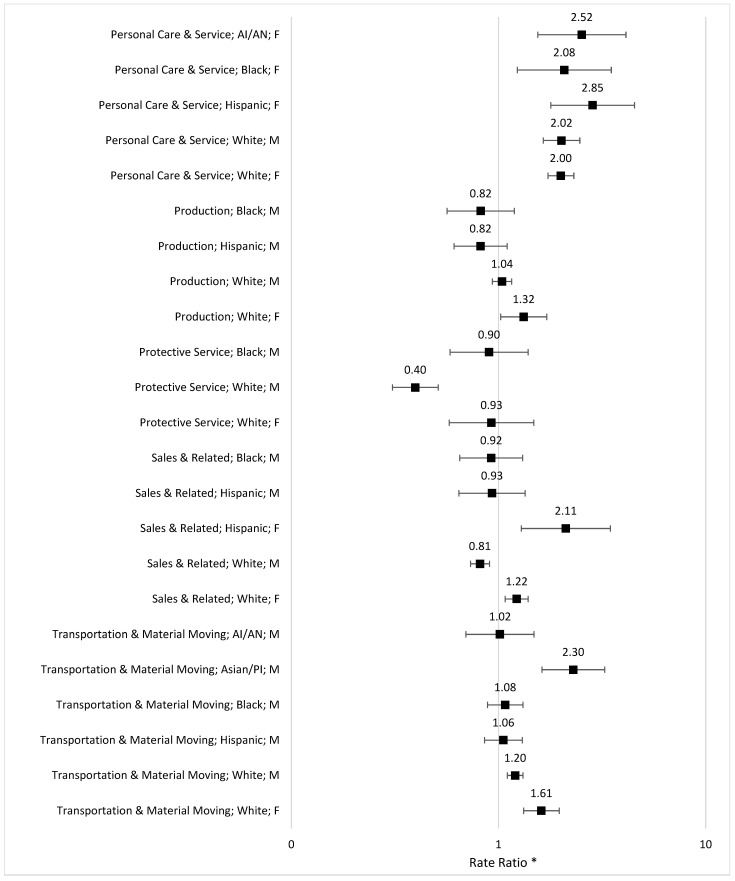
Drug overdose rate ratios and 95% CI for Washington workers stratified by occupation group, race/ethnicity, and sex, 2014–2023. * Drug overdose rate ratios comparing age-adjusted rates among workers in specific occupations to the baseline age-adjusted rate for all workers in the same race/ethnicity and sex group. 95% CI = 95% Confidence Interval; AI/AN = American Indian/Alaskan Native; PI = Pacific Islander.

We display similar results for drug overdose deaths in Figure 4. There were fewer strata suppressed here due to larger numbers of deaths (n = 10,695) as compared to suicides in Figure 3 (n = 7306). As with suicides, we identified increased risk (2–3 times higher) of drug overdose death for males across all race/ethnicity groups, as well as White females, Figure 4. Black males had a nearly five times higher drug overdose rate when working in Arts, Design, Entertainment, and Media occupations compared to other Black male workers. As with suicide, we report an increased risk for White females working in Healthcare Support occupations as compared to all White female workers, and no change for White males in those occupations. Finally, we report increased drug overdose death risk among several demographic groups working in Food Preparation & Serving occupations—Asian/Pacific Islander males, White males, and White females, as well as groups working in Personal Care & Services occupations—American Indian/Alaskan Native females, Black females, Hispanic females, White females, and White males. This is not an exhaustive list of important findings within these data. We highlight only a few to aid in the interpretation of the results and to emphasize certain patterns.

## 4. Discussion

Prevention of suicide and drug overdose deaths are public health priorities [15,39,40,41,42]. This work describes suicide and drug overdose mortality risk among the working-age population by industry and occupation while addressing the differences in multiple worker demographic factors, filling a gap in the current research, as few others have described [18,19,43]. Further, we provide Washington State-specific data of critical importance for informing potential priorities at the state level and providing actionable data for industries, worker groups, and local public health programs. We also demonstrate repeatable methods with data that are available to all states. Finally, these results add to the body of literature that further identifies disparities in suicide and overdose risk by industry and occupation. Most importantly, our results verify that the disparity in risk by some industry and occupation groups is not solely due to the segregation of work by age, sex, and race/ethnicity.

The disparities we observed for suicide and drug overdose mortality across demographics are consistent with national findings [2,44,45]. Race is a social construct, not a biological one; thus, race serves to identify the likely presence of upstream risk factors for suicide and drug overdose, such as historical underfunding in health and mental health programs in certain communities, historical trauma, historical or ongoing systemic racism, or stigma amongst certain communities for harm reduction usage [46,47]. We report that American Indian/Alaskan Natives and Whites had higher than average rates of suicide and drug overdose death, while Blacks had higher than average rates of drug overdose mortality, supporting previously reported findings [2,44,45]. Alternatively, crude rates of suicide were lowest among Hispanics, Blacks, and Asian/Pacific Islanders, aligning with previous research that has identified protective factors for suicide in these groups [48,49,50]. Drug overdose rates were lowest among Hispanics and Asian/Pacific Islanders, suggesting some of the same protective factors influencing suicide risk may apply to preventing drug overdose deaths as well.

The industries and occupations most at risk for overdose fatality were similar to what other studies have found [18,30,51,52]. In addition to supporting the current classification of high-risk industries and occupations, we were able to identify how the risk associated with worker sex and race/ethnicity is modified when working within certain industries and occupations. The Construction & Extraction occupations employ a high proportion of males (>95%), which increases suicide and drug overdose death rates compared to occupations employing a greater proportion of females [24]. However, our results demonstrated additional risk for males. Males from all race/ethnicity groups had higher suicide and drug overdose death rates when working in Construction & Extraction occupations as compared to all other occupations combined. Our findings also detail how this effect varied by sex and race/ethnicity group. For example, American Indian/Alaskan Native males have the highest age-adjusted suicide rates within the Construction & Extraction occupation; however, suicide risk among Asian/Pacific Islander males who work in the Construction & Extraction occupation increased the most from their demographic baseline rate (had the highest rate ratio).

Construction workers have been shown to have many risk factors that could impact risk of anxiety and depression, including inappropriate safety measures, high workload, tougher financial situations, and lack of reward from work [53]. All of these factors may help explain the increased rates of both suicide and drug overdose mortality in construction as well as similar fields experiencing stressful work environments, low pay or unemployment, lower workplace safety, and/or longer work hours [54,55,56]. Construction workers also have increased rates of musculoskeletal disorders and chronic musculoskeletal pain, putting them at increased risk of opioid use disorder and opioid-related overdose mortality [57,58,59,60]. As noted above, the Asian/Pacific Islander males had significantly higher rate ratios than any other race/ethnicity group, aside from Black males. It is unclear whether work in construction and extraction increased risk for Asian/Pacific Islander men due to the impact of this occupation alone or if there are additional compounding risks for Asian/Pacific Islander men in this occupation. Further research into the intersection of Asian/Pacific Islander men among similar occupations is recommended to better understand these findings. Particularly, using the cultural model of suicide would be particularly helpful in identifying cultural stressors, meanings, and suicidal ideation among this group [61].

Interestingly, we also noticed that Hispanic males in the Farming, Fishing, & Forestry occupations have significantly lower drug overdose mortality rates compared to Hispanic males overall, highlighting a potential that protective factors exist for Hispanic males in these occupations. Research has posited protective factors among Hispanics living in America, like strong religious and cultural beliefs, such as familism and collectivism; however, more specific research may help understand potential strength or frequency of protective factors among Hispanics working within the Farming, Forestry, & Fishing occupations [62,63]. Working in Farming, Fishing, & Forestry did not change suicide risk for Hispanic males, but more than doubled the risk for White males. This varied change in risk may reflect the different roles typically assumed for White males versus Hispanic males in these occupations. White males are much more likely to be the producers and/or owners who have decision-making responsibility and are exposed to unique financial pressures, versus farmworkers, who are more likely to be Hispanic [64,65]. Broadly, these results may be explained in part by the Healthy Immigrant Effect (HEI), whereas US immigrants seem to have mental health advantages as compared to their non-immigrant counterparts [66]. This phenomenon may be contributing to the lower suicide and drug overdose rate ratios observed. Additional factors for workers in Farming, Fishing, & Forestry occupations, such as H-2A visa status and work among undocumented immigrants, may also be influencing these relationships with suicide and/or drug overdose risk in unknown ways. Future research should investigate the relationship between immigration status and suicide and/or overdose risk.

Previous research also identified workers in the Arts, Design, Entertainment, Sports, & Media occupations to have higher rates of suicide and drug overdose death compared to other occupations [19,52,67]. Researchers have debated additional reasons for increased risk of suicide and drug overdose deaths within this occupation [68]. Research specifically on workers in the music industry identified potential risk factors including financial precarity; history of physical, emotional, or sexual abuse; and/or unique performance-related stressors [68]. These factors may expand to other detailed occupations within the broader Arts, Design, Entertainment, Sports, & Media occupation group. Our results suggest that the work-related risk factors within these occupations differentially affect risk across race/ethnicity groups. The interplay of work-related risk factors and systemic inequities by race/ethnicity warrants further research, especially among Black males who were close to five times more likely to die by drug overdose as compared to other Black male workers.

Similar to others, we identified Food Preparation & Serving to be a higher risk occupation for suicide and drug overdose death [18,19]. It has been hypothesized that increased risk of suicide in the Food Preparation & Serving occupations may be related to increased risk factors like alcohol consumption, occupational stress, and sleep disturbances [69]. Suicide risk was only increased among White males and females, while drug overdose risk was increased among the following groups: Asian/Pacific Islander males, Black males, Hispanic males, and White males and females.

Like others, we found the Protective Service occupations, which include law enforcement officers and firefighters, to have higher than average crude suicide rates [18,19]. However, our work adds more context to this finding. Our results indicate that the suicide rate among White males working in Protective Service occupations is similar to the rate among all White male workers. Even so, previous research has shown there are known work exposures, such as chronic stress inducing hopelessness, anxiety, and depression; witnessing and experiencing trauma with a higher prevalence of PTSD; and less supportive work environments that suggest protective service occupations may be at higher risk of suicide [70,71,72]. There are also hypothesized protective factors associated with work in Protective Service occupations, including workplace social support, family connectedness, and friendship networks in the community [73].

Other studies that investigate suicide and drug overdose mortality typically focus on and report on only the industries and occupations with which there is the highest risk, but rarely do investigators highlight industries and occupations with lower risk. We found that after adjusting for age and stratifying by sex and race/ethnicity, the Education, Training, & Library occupations had far lower rates of both suicide and drug overdose mortality compared to the overall rates of suicide and overdose within a racial/ethnic and sex group overall. Other research has highlighted potential reasons as to what socioeconomic factors are associated with decreased risk for suicide and drug overdose mortality, including but not limited to higher educational attainment, job safety, and strong social relationships [74,75,76]. These factors may be present more often among the education profession and thus decrease suicide and drug overdose risk [74].

### Strengths and Limitations

This study has several strengths and adds important context to the understanding of suicide and drug overdose death risk by industry and occupation. Most importantly, we compare death rates of workers to their respective baseline rates based on sex and race/ethnicity. Stratification allowed us to address the possible effect modification and confounding of these covariates that may influence both someone’s suicide and overdose risk, as well as their probability of working in a specific industry or occupation [25]. We also provided rates and rate ratios for all industry and occupation groupings, and not just those with high rates, which allowed us to identify industries and occupations at lower risk. The relationships we have identified in this research generate hypotheses and provide direction for future work in understanding how the effect of working in a specific industry or occupation is modified by racial/ethnic structural inequities. Finally, providing Washington State-specific data allows decision-makers at the state level to develop policies for prevention for Washington workers; however, we also provide a generalizable methodology for other states. All states have access to their state-specific numerator and denominator data used in this study.

This study does have some limitations. First, we only provide information for 2-digit NHIS groupings of industry and occupation. This may result in a broad interpretation of risk that is driven by a small subset of workers within the industry or occupation. The impact of industry and occupation on suicide and drug overdose risk deserves further exploration, and analyses of more specific industry and occupation groupings are needed, but are beyond the scope of this project. Second, using ‘usual’ industry and occupation from the death certificate may have resulted in some misclassification for decedents working in multiple industries or occupations during their lifetime; however, Luckhaupt et al. report good concordance between current and usual occupation, especially among adults less than 65 years of age [32]. The death certificate data includes residents in Washington State regardless of immigration status; however, the ACS may not collect information on undocumented workers as comprehensively, so we would expect rates to be overestimated among industries and occupations with more undocumented workers. Additionally, suicide mortality has been shown to be underreported due to religious and social stigma associated with this specific cause of death, leading to suicide deaths being classified as having “unknown intent” [10]. Suicide classification has also become increasingly complex as drug overdose deaths increase with unknown intent. Misclassification of suicide is possible in our study; however, we included deaths of unknown intent to minimize the effect. Further, our results do not identify risk and protective factors for suicide and overdose mortality across the entirety of the social-ecological model, including many non-occupational and occupational contributors that might be available through alternative data systems like the National Violent Death Reporting System [15]. For example, income, education, history of mental illness, access to firearms, and access to opioids or other drugs. This study is unable to address the temporality of risk factors for suicide and drug overdose death in relation to industry and occupation. It is unclear if risk increases after working within certain industries or occupations, or if the factors that increase suicide or drug overdose death risk also influence which industry and/or occupation a worker is employed in. There are competing theories about whether socioeconomic circumstances influence mental health, ‘Social Causation’, or if a predisposition towards mental illness limits opportunity and leads people to drift towards lower Socio-Economic Status (SES), ‘Social Selection/Drift’ [77]. In *The Wiley Blackwell Encyclopedia of Health, Illness, Behavior, and Society*, Mossakowski summarizes evidence for both theories and concludes that “both social causation and social selection contribute to disparities in SES and mental health status” and that the “direction and strength of the relationship between disadvantaged SES and mental illness can vary by type of mental disorder and indicator of SES.” Like Mossakowski, we hypothesize that some suicide and drug overdose risk factors are present before employment, while employment in specific industries or occupations adds new risks or exacerbates risks already present. Our results were limited by sample size, and many stratified rates were suppressed. This undoubtedly restricts the interpretation of findings among some specific demographic groups, which may have increased or decreased risk of suicide or fatal drug overdose, especially among female workers. Finally, the data available to us aggregates race and ethnicity into several broad groups, and we recognize that additional detail describing the heterogeneity of these racial and ethnic groups could further reveal more defined populations with suicide and drug overdose risk, particularly among the Asian/Pacific Islander group [78]. Wide confidence intervals from small sample sizes that identify ‘null’ findings do not imply that there are not potential important effects among these groups regarding suicide and drug overdose risk.

## 5. Conclusions

There is a strong body of literature characterizing suicide and drug overdose death risks. To our knowledge, this study is the first of its kind to evaluate how the commonly cited disparities across gender and race/ethnicity change depending on industry and occupation. The effects of historical and structural sexism and racism are apparent in the occupational segregation that persists today in the US labor force. Although we are unable to detail the causal pathway for workers across a lifetime, this work confirms that occupational segregation isn’t the sole driver of suicide and drug overdose disparities by industry and occupation. Instead, we see unique disparities of mortality across sex and race/ethnicity within specific industries and occupations. This study also expands our understanding of suicide and drug overdose mortality burden across industry and occupation in Washington State for specific sex and race/ethnicity groups. Understanding how the risk of suicide and drug overdose deaths relates to occupation and industry is critical in efforts to reduce mortality due to these causes. To more accurately describe this relationship, stratifying rates by potential effect modifiers (sex and race/ethnicity) and adjusting for age was imperative. Further understanding these multifactorial relationships should be a focus of future research to better understand the risk for suicide and drug overdose across industry and occupation. Additionally, understanding work-related factors like low wages, long working hours, risk of injury, and the increase of low-security jobs should continue to be areas of focus for suicide and drug overdose prevention among certain occupations and industries [54,55,56,79]. Although the cycle of causes and effects of risk factors for suicide and drug overdose mortality is complex, understanding where potentially at-risk people are working may lend insight into more impactful suicide and drug overdose prevention programs for coworkers, employers, health professionals, and the government. Aims to improve workplace safety standards to reduce chronic injury, increase wages and labor protections, and assess workplace culture regarding suicide and drug overdose risk factors should be of high importance. Additional research is needed to understand the relationships between employment in specific industries and occupations and the risk of suicide or overdose over time, as well as the specific impact work-related injuries may have on the risk of suicide or overdose mortality.

## Figures and Tables

**Table 1:** Crude mortality rates for suicide, drug overdose, and all other deaths by demographic variables among Washington residents aged 16–65, 2014–2023.

	Suicides * (n = 9581)		Drug Overdose Deaths * (n = 14,239)		All Other Deaths (n = 116,252)	
	Rate per 100,000 residents (95% CI)	%	Rate per 100,000 residents (95% CI)	%	Rate per 100,000 residents (95% CI)	%
Total	20.8 (20.2, 21.4)	100%	30.9 (30.0, 31.7)	100%	252.0 (246.4, 257.6)	100%
Sex						
Female	10.3 (9.8, 10.7)	24%	20.3 (19.6, 21.0)	32%	201.6 (198.0, 205.2)	39%
Male	30.9 (29.8, 32.1)	76%	41.1 (39.6, 42.5)	68%	300.6 (291.8, 309.7)	61%
Age Group						
16–24	18.9 (17.7, 20.2)	15%	14.4 (13.4, 15.5)	8%	43.3 (41.0, 45.8)	3%
25–34	19.4 (18.4, 20.5)	21%	28.4 (27.1, 29.8)	21%	63.0 (60.5, 65.6)	6%
35–44	19.9 (19.0, 20.9)	20%	33.6 (32.4, 34.9)	23%	118.0 (115.2, 120.8)	10%
45–54	22.9 (21.9, 23.9)	21%	38.2 (36.8, 39.5)	24%	299.3 (294.3, 304.3)	23%
55–65	22.8 (21.8, 23.8)	22%	37.6 (36.3, 38.9)	24%	746.1 (737.0, 755.3)	58%
Race/Ethnicity						
AI/AN	51.4 (45.2, 58.5)	3%	142.6 (131.3, 154.8)	5%	750.6 (715.8, 787.2)	3%
Asian/PI	12.1 (11.2, 13.1)	6%	8.7 (7.9, 9.5)	3%	137.1 (133.2, 141.2)	6%
Black	18.9 (16.9, 21.2)	4%	62.2 (57.8, 66.8)	9%	363.7 (345.8, 382.6)	6%
Hispanic	12.3 (11.4, 13.3)	8%	20.0 (18.8, 21.2)	9%	141.7 (137.3, 146.3)	8%
White	23.4 (22.7, 24.1)	79%	32.8 (31.9, 33.8)	75%	276.8 (271.1, 282.7)	77%

* Suicide and drug overdose are not mutually exclusive categories. Total number of deaths n = 138,370 (see Figure 1); suicide and/or drug overdose deaths n = 22,118, and all other n = 116,252. AI/AN = American Indian/Alaskan Native; PI = Pacific Islander; 95% CI = 95% Confidence Interval.

**Table 2:** Crude mortality rates of suicide and drug overdose by industry group among Washington workers, 2014–2023.

Suicide		Drug Overdose	
Industry Group	Mortality Rate per 100,000 Workers (95% CI)	Industry Group	Mortality Rate per 100,000 Workers (95% CI)
Mining, Oil, & Gas Extraction	56.0 (36.1, 86.9)	Construction	84.1 (80.2, 88.3)
Construction	45.6 (42.9, 48.5)	Mining, Oil, & Gas Extraction	48.7 (30.3, 78.3)
Transportation & Warehousing	30.4 (27.8, 33.2)	Arts, Entertainment, & Recreation	45.3 (41.0, 50.1)
Arts, Entertainment, & Recreation	28.9 (25.5, 32.7)	Ag, Forestry, Fishing, & Hunting	44.8 (40.7, 49.3)
Utilities	27.1 (21.6, 34.1)	Accommodation & Food Services	41.9 (39.1, 44.8)
Other Services (except Public Admin.)	25.6 (23.3, 28.2)	Other Services (except Public Admin.)	41.6 (38.5, 44.9)
Ag, Forestry, Fishing, & Hunting	24.2 (21.3, 27.5)	Admin., Support, Waste Mgmt, & Remediation	34.9 (31.9, 38.1)
Admin., Support, Waste Mgmt, & Remediation	21.1 (18.8, 23.6)	Transportation & Warehousing	33.3 (30.6, 36.2)
Manufacturing	20.6 (19.1, 22.2)	**All Industries average**	26.8 (26.0, 27.7)
**All Industries average**	19.3 (18.7, 20.0)	Manufacturing	23.2 (21.6, 24.9)
Accommodation & Food Services	18.0 (16.4, 19.8)	Retail Trade	21.4 (20.0, 22.9)
Public Administration	17.6 (15.8, 19.6)	Health Care & Social Assistance	19.1 (17.9, 20.4)
Prof., Scientific, & Tech Serv.	17.5 (16.1, 18.9)	Real Estate, Rental, & Leasing	17.6 (14.8, 21.0)
Real Estate, Rental, & Leasing	16.8 (14.1, 20.1)	Utilities	15.7 (11.6, 21.2)
Information	15.4 (13.0, 18.3)	Information	13.0 (10.8, 15.7)
Retail Trade	14.8 (13.6, 16.0)	Wholesale Trade	11.2 (9.2, 13.5)
Health Care & Social Assistance	13.1 (12.1, 14.2)	Prof., Scientific, & Tech Serv.	11.2 (10.1, 12.3)
Finance & Insurance	12.9 (11.0, 15.1)	Public Administration	10.7 (9.3, 12.3)
Wholesale Trade	8.6 (6.9, 10.7)	Finance & Insurance	10.4 (8.7, 12.5)
Education	6.9 (6.0, 7.9)	Mgmt of Companies & Enterp.	S
Mgmt of Companies & Enterp.	S	Education	5.2 (4.5, 6.1)

95% CI = 95% Confidence Interval; Prof., Scientific, & Technical Serv. = Professional, Scientific, & Technical Services; Mgmt of Companies & Enterp. = Management of Companies and Enterprises; Admin., Support, Waste Mgmt, & Remediation = Administration, Support, Waste Management & Remediation; S = Suppressed. Bolded all industry average rates are included to show which specific industry rates fall above or below overall rates.

**Table 3:** Crude mortality rates for suicide and drug overdose by occupation group among Washington workers, 2014–2023.

Suicide		Drug Overdose	
Occupation Group	Mortality Rate per 100,000 Workers (95% CI)	Occupation Group	Mortality Rate per 100,000 Workers (95% CI)
Construction & Extraction	54.9 (51.4, 58.7)	Construction & Extraction	102.6 (97.3, 108.0)
Installation, Maintenance, & Repair	40.0 (36.4, 44.0)	Installation, Maintenance, & Repair	50.9 (46.7, 55.4)
Arts, Design, Entertainment, Sports & Media	29.0 (25.6, 32.8)	Farming, Fishing, & Forestry	49.8 (44.6, 55.5)
Transportation & Material Moving	27.7 (25.6, 30.0)	Food Preparation & Serving	43.1 (40.1, 46.4)
Protective Service	26.4 (22.8, 30.5)	Transportation & Material Moving	39.3 (36.7, 42.0)
Production	23.7 (21.4, 26.1)	Building & Grounds Cleaning & Maintenance	38.9 (35.4, 42.7)
Farming, Fishing, & Forestry	23.6 (20.2, 27.5)	Arts, Design, Entertainment, Sports & Media	38.3 (34.4, 42.7)
Life, Physical, & Social Sciences	20.5 (16.7, 25.2)	Personal Care & Services	33.6 (30.4, 37.1)
Architecture & Engineering	20.0 (17.4, 23.1)	Production	31.5 (28.9, 34.4)
**All Occupations average**	19.3 (18.6, 19.9)	Healthcare Support	28.2 (25.3, 31.5)
Building & Grounds Cleaning & Maintenance	19.0 (16.7, 21.6)	**All Occupations average**	27.1 (26.3, 28.0)
Personal Care & Services	18.5 (16.3, 21.1)	Sales & Related	23.8 (22.1, 25.5)
Food Preparation & Serving	16.9 (15.2, 18.8)	Protective Service	21.1 (18.0, 24.8)
Sales & Related	16.5 (15.2, 18.0)	Community & Social Services	16.6 (13.7, 20.2)
Management	15.3 (14.2, 16.5)	Office & Administrative Support	15.7 (14.5, 17.1)
Legal	14.1 (10.6, 18.7)	Life, Physical, & Social Sciences	14.7 (11.5, 18.7)
Computer & Mathematical	14.0 (12.4, 15.7)	Healthcare & Technical	13.2 (11.7, 14.9)
Healthcare Support	13.7 (11.7, 16.0)	Management	12.9 (11.9, 14.0)
Community & Social Services	13.5 (10.9, 16.7)	Architecture & Engineering	10.9 (9.0, 13.2)
Healthcare & Technical	13.3 (11.8, 15.0)	Legal	10.2 (7.3, 14.3)
Office & Administrative Support	11.6 (10.6, 12.7)	Business & Financial Operations	8.1 (7.0, 9.5)
Business & Financial Operations	9.7 (8.5, 11.2)	Computer & Mathematical	7.7 (6.6, 9.1)
Education, Training, & Library	6.9 (5.9, 8.1)	Education, Training, & Library	5.5 (4.6, 6.6)

95% CI = 95% Confidence Interval. Bolded all occupation average rates are included to show which specific occupation rates fall above or below overall rates.

## Data Availability

Denominator data for rate calculations presented in the study are openly available from the Integrated Public Use Microdata Series (IPUMS) USA. For numerator data used to identify suicide and drug overdose fatalities, restrictions apply to the availability of these data. Data were obtained from the Washington State Department of Health under a data sharing agreement.

## Supplementary Materials

The following supporting information can be downloaded at: https://www.mdpi.com/article/10.3390/ijerph23060699/s1, Table S1: Age-adjusted suicide rates among female workers in Washington State stratified by occupation group and race/ethnicity, 2014–2023; Table S2: Age-adjusted drug overdose rates among female workers in Washington State stratified by occupation group and race/ethnicity, 2014–2023; Table S3: Age-adjusted suicide rates among male workers in Washington State stratified by occupation group and race/ethnicity, 2014–2023; Table S4: Age-adjusted drug overdose rates among male workers in Washington State stratified by occupation group and race/ethnicity, 2014–2023; Table S5: Age-adjusted suicide rates among female workers in Washington State stratified by industry group and race/ethnicity, 2014–2023; Table S6: Age-adjusted drug overdose rates among female workers in Washington State stratified by industry group and race/ethnicity, 2014–2023; Table S7: Age-adjusted suicide rates among male workers in Washington State stratified by industry group and race/ethnicity, 2014–2023; Table S8: Age-adjusted drug overdose rates among male workers in Washington State stratified by industry group and race/ethnicity, 2014–2023.

## Abbreviations

The following abbreviations are used in this manuscript:

ACS	American Community Survey
ICD-10	International Classification of Diseases, 10th Revision
CDC	Centers for Disease Control and Prevention
NOMS	National Occupational Mortality Surveillance
NIOSH	National Institute for Occupational Safety and Health
NIOCCS	NIOSH Industry and Occupation Computerized Coding System
NHIS	National Health Interview Survey
CI	Confidence Intervals
RSE	Relative Standard Error
AI/AN	American Indian/Alaskan Native
PI	Pacific Islander
CSTE	Council of State and Territorial Epidemiologists
IPUMS	Integrated Public Use Microdata Series
